# Pulse-Duration-Sensitive High Harmonics and Attosecond Locally Chiral Near Fields from a Chiral Topological Weyl Semimetal

**DOI:** 10.1021/acs.nanolett.6c00913

**Published:** 2026-07-20

**Authors:** Alba de las Heras, Zhengwei Nie, Subhojit Pal, Umberto De Giovannini, Ofer Neufeld, Angel Rubio

**Affiliations:** † 16779 Max Planck Institute for the Structure and Dynamics of Matter, Center for Free-Electron Laser Science, Hamburg 22761, Germany; ‡ Università degli Studi di Palermo, Dipartimento di Fisica e Chimica − Emilio Segrè, Palermo 90123, Italy; § 18998 Technion Israel Institute of Technology, Faculty of Chemistry, Haifa 3200003, Israel; ∥ Center for Computational Quantum Physics, The Flatiron Institute, New York, New York 10010, United States

**Keywords:** Attosecond science, Quantum topological materials, Chirality; High harmonic generation, Ultrafast electron dynamics

## Abstract

High harmonic generation (HHG) in solids results from an interplay between intraband acceleration and electron–hole recombination driven by a high-intensity laser pulse. Here, we theoretically reveal that the driving pulse duration can play a major role in extending HHG to higher photon energies by promoting higher conduction band excitations. The effect is present in a conventional semiconductor as Si, restricted in a large-gap insulator as MgO, and most prominent in RhSi, a prototypical chiral Weyl semimetal presenting numerous band crossings. Further, we elucidate the HHG selection rules in RhSi required for the synthesis of attosecond locally chiral near fields. The chiral crystal structure enables the generation of a 3D electric field exhibiting an asymmetric instantaneous torsion on attosecond time scales. Our findings motivate future experiments in chiral Weyl semimetals to track high-energy band crossings and in situ locally chiral light, advancing prospects for compact extreme-ultraviolet sources, chiral sensing and ultrafast optoelectronics.

## Graphical abstract

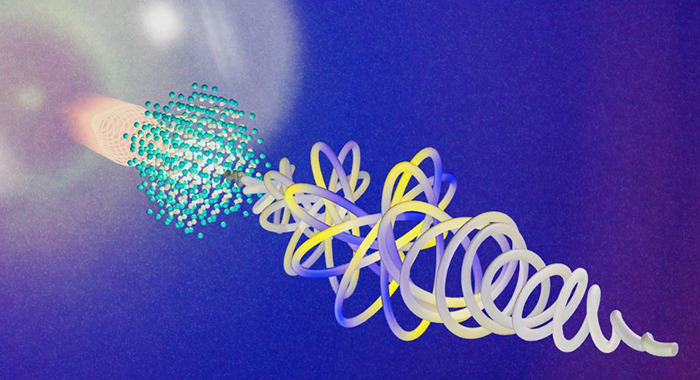

High harmonic generation (HHG) is paramount for the synthesis of attosecond pulses and the traceability of electron dynamics.^1–6^ Dilute gases composed of atomic or molecular species have long been the standard targets for HHG, until the first observation in a bulk crystal.^7^ Nowadays, HHG from quantum topological materials is an extremely active research line, covering Dirac^8–10^ and Weyl semimetals,^11–19^ as well as topological insulators.^20–22^ Chiral Weyl semimetals are particularly attractive due to the combined handedness in the crystal lattice and in the electronic Weyl fermions,^23–25^ but their potential has not yet been exploited in HHG.

HHG is characterized by the presence of nonperturbative plateaus ending at a cutoff energy.^7,26,27^ However, the well-known picture of HHG in atoms and molecules, based on the three steps of (i) tunnel ionization, (ii) acceleration during the electron real-space excursion and (iii) recombination,^28,29^ is more complex in solids. HHG in solids results from intraband and interband contributions.^30^ Intraband HHG originates from the nonlinear electron motion within a band, whereas the interband pathway requires (i) electron excitation into a conduction band, (ii) displacement in momentum space and (iii) electron–hole recombination.^31^ The understanding of interband and intraband contributions in solid-state HHG enables the retrieval of quantitative information on the band structure,^27,32–37^ crystal symmetries,^38,39^ and subcycle carrier dynamics.^40,41^ The energy cutoff in HHG is typically ruled by interband dynamics, so that the maximum photon energy is given by the maximum energy of electron–hole recombinations.^42^ Multiple plateaus are known to emerge due to high-energy transitions from higher conduction bands.^27,42–47^ A subtle parameter in HHG from solids is the driving pulse duration, which has been predicted in 1D models to be crucial in the emergence of secondary plateaus.^45^ Nevertheless, a rigorous pulse duration analysis on solid-state HHG has not yet been reported from experiments or simulations.

Crystals offer an additional opportunity to imprint their symmetries on the high-frequency emission, departing from the conventional approach of tailoring the driving field to control the properties of HHG.^48–50^ For instance, harmonic beams can be produced with a high ellipticity in solids,^51–53^ as an alternative to the schemes proposed in gas targets.^54–57^ Equally important, the absence of certain symmetries enables richer phenomena, such as the circular photogalvanic effect in materials lacking inversion symmetry.^58–63^ The absence of mirror symmetry, known as chirality, is vividly studied in molecular systems^64–69^ because enantiomer sensitivity is critical in biomedical applications and in catalytic processes for chemical design. HHG can similarly probe the structural chirality of crystals.^70,71^ However, chiral crystals have not been investigated as a way to imprint local chirality in HHG. The key concept of locally chiral light is to engineer a nonsuperimposable mirror-image electric field microscopically for a more efficient dipolar chiral interaction with matter.^72–76^ Still, the generation and characterization of locally chiral light remain experimentally challenging, due to the requirements of high spectral control and near-field detection, and its interaction and emergence in chiral solids have not yet been explored.

Here, we investigate HHG in RhSi—a well-established chiral Weyl semimetal^23–25^ —using time-dependent density-functional theory (TDDFT). The double aim is (i) to unveil the role of pulse duration in solid-state HHG, and (ii) to demonstrate that the structural chirality maps into locally chiral HHG. In the first results part, our *ab initio* calculations show that the duration of the driving laser pulse substantially influences the quantum pathways of electron excitation to higher conduction bands and, in consequence, the cutoff energy in HHG. The effect is paramount in RhSi due to its high multiband coupling, moderate in Si, and severely suppressed in the large-gap insulator MgO. In the second part, we explore a complementary outcome of HHG from chiral Weyl semimetals. We identify HHG selection rules in RhSi to synthesize locally chiral near fields. By exploiting the strong-field interaction of a circularly polarized driver with the chiral crystal structure, it is possible to engineer a 3D electric field vector presenting an asymmetric instantaneous torsion on attosecond time scales. Such locally chiral polarization varying on hundreds of attoseconds sets a new route to induce and probe attosecond chiral excitations within dipolar interactions. Overall, our work establishes chiral Weyl semimetals as a promising material family for observing and manipulating out-of-equilibrium phenomena.

We first consider a laser pulse with a central photon energy of ∼1 eV and linear polarization along the [111] crystal direction. In Figure 1, we compare driving full-pulse durations of 4, 12, and 20 optical cycles. The driving pulse durations are chosen to address typical experimental full-width half-maximum intensity values of 5.4, 17.4, and 29.0 fs, respectively. Each panel presents the band structure, the high harmonic spectrum, and the change of the electronic density of occupied states, |ΔDOOS| in eq 1, for (A) RhSi, (B) Si and (C) MgO. The inset shows the crystal unit cell and the geometry of the incident electric field. To produce comparable HHG responses, the driving peak intensity is set to 1 TW/cm^2^ in RhSi and Si, whereas 3 TW/cm^2^ is chosen for MgO due to its larger bandgap.

**Figure 1:**
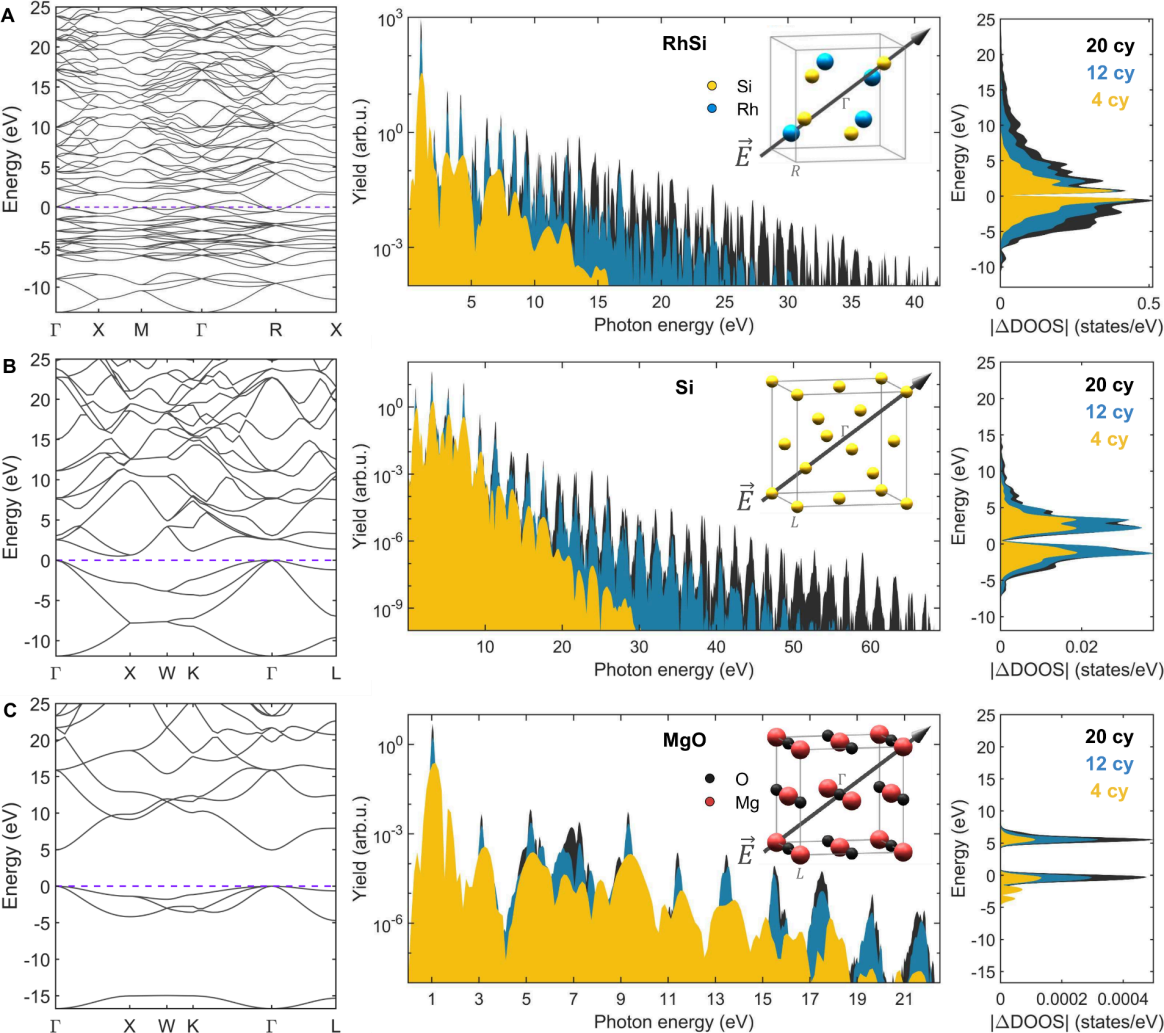
Pulse duration dependence of high harmonic generation in bulk crystals. Computed band structure (left), high harmonic spectrum (center) and change of the density of occupied states (right) for three different materials: (A) RhSi, (B) Si and (C) MgO. The Fermi energies are indicated at 0 eV (dashed violet line). The full duration of the driving laser pulse is set to 4 (yellow), 12 (blue) and 20 (black) optical cycles. In RhSi and Si, longer durations of the driving pulse are associated with the occupation of higher-energy states and a substantial extension of the emission toward higher photon energies. The insets show the unit cell and the direction of the linearly polarized electric field.

Figure 1 shows an extreme sensitivity of the HHG cutoff in RhSi and Si to the pulse duration. In Si, the cutoff is extended from ∼ 25 to ∼ 60 eV when increasing the pulse duration from 4 to 20 cycles, while keeping constant the peak intensity and other driving parameters. The harmonic yield follows a characteristic multiplateau behavior, presenting abrupt stepped decays at ∼7, 26, 43, and 59 eV. Consequently, the highest plateau (at 43–59 eV) is emitted with a reduction in the harmonic yield of 8 orders of magnitude, compared to the standard plateau (at <7 eV). In RhSi, the cutoff extension expands from ∼7 to ∼35 eV. Even though the different multiplateau structures are difficult to discern in RhSi, the HHG cutoff extension emerges as a clear signature, with a smoother decay of the harmonic yield spanning a 3-order-of-magnitude reduction. The higher microscopic yield of RhSi compared to Si or MgO suggests that RhSi should be a promising material for observing the energy cutoff extension in experiments. While the energy cutoff is expected to depend on the driving photon energy and peak field amplitude,^7,77^ the influence of the pulse duration implies a progressive electron ascension through the conduction bands during the interaction with the laser pulse.^45^

As an opposite case, the high harmonic spectrum in MgO (Figure 1C) does not present a notorious dependence of the energy cutoff on the pulse duration. We recognize the standard effects of longer pulses, such as the harmonic peak narrowing and the yield enhancement coming from the periodicity of more recombination events. In the following, we aim to elucidate the origin of the cutoff extension and the material characteristics that favor or hinder this mechanism. To evaluate the electron rearrangement induced by the laser pulse, we define the change in the density of occupied states by

$$|\Delta \mathrm{DOOS}\left(E\right)|=\left|\underset{k,n}{\sum }\left[f_{k,n}\left(t=\tau_{\mathrm{pulse}}\right)-f_{k,n}\left(t=0\right)\right]w_{k}\delta \left(E-E_{k,n}\right)\right|$$ 1

where *f*_*k*,*n*_(*t*) are the occupation coefficients of each eigenstate at time *t*, *w*_*k*_ the k-point weights, *E* the energy variable, *E*_*n*,*k*_ the eigenvalues, τ_pulse_ the full duration of the driving laser pulse and *t* = 0 the initial time before the laser pulse. The function |ΔDOOS­(*E*)| is plotted in the right column of Figure 1 for different driving pulse durations in RhSi, Si and MgO. Longer pulses promote electron population to high-energy states in RhSi and Si, but not in MgO. The occupations in MgO show that the valence bands are only coupled to the first conduction band. This dismantles the possibility of increasing the energy of electron–hole recombinations that is required for higher-order harmonic plateaus.

The correlation between the HHG extension and a higher occupation of high conduction bands with compatible energy transitions points to a mechanism of *“excited ladder electrons”*. During the interaction of the laser pulse, the progressive electronic excitation to higher conduction bands allows for higher energy electron–hole recombinations. The large bandgap of MgO prevents a strong coupling to higher energy states, since the dipole matrix elements are inversely proportional to the energy difference of the transition. It requires substantially higher intensities to observe a secondary plateau.^47^ Conversely, the high density of states and intricate network of band crossings observable in the band structure of RhSi favors the multiband coupling and the ascent of electrons to high-energy states even for lower driving intensities. This is in consonance with the higher harmonic yield in RhSi at high photon energies. Silicon can be interpreted as an intermediate situation, where the smaller bandgap allows the multiband coupling but it is not as favored as in RhSi. This is also compatible with the reduced yield obtained in the HHG spectra in Si.

Even if high-energy excitation pathways are allowed by the material characteristics and the laser parameters, the occupation of high-energy states requires several interaction cycles.^45^ The mechanism of excited ladder electrons is cumulative, so the population of higher-energy states increases for longer driving pulses. For a better insight into the progressive buildup of HHG, we continue the analysis of the emitted radiation. In Figure 2, the time-resolved spectrogram of HHG in RhSi, Si and MgO provides information about the photon energy emission as a function of time. Again, we compare driving pulse durations of 4 (left column), 12 (center) and 20 (right column) optical cycles. On the one hand, in RhSi and Si, higher photon energies are emitted as the number of optical cycles increases. After the initial cycles, the energy cutoff scales linearly with the interaction time (see Figure 2B,C,E,F). More importantly, the high energy emission arises only at longer times, requiring an initial activation consistent with the excitonic electron ladder mechanism. The emission also persists even at the end of the laser pulse, until the eventual vanishing due to subsequent decoherent effects not included in the simulations. On the other hand, the emission in MgO shows a similar behavior for all pulse durations (Figures 2G–I), which resembles the usual HHG in atoms. In MgO, the photon energies around the energy cutoff are emitted around the central times of the pulse when the peak intensity is higher. This reveals the different HHG emission from a few-band-coupling in MgO or a high-multiband-coupling in Si and RhSi. Indeed, the time-resolved spectrograms enable us to retrieve the time required for high-energy electron–hole recombination.

**Figure 2:**
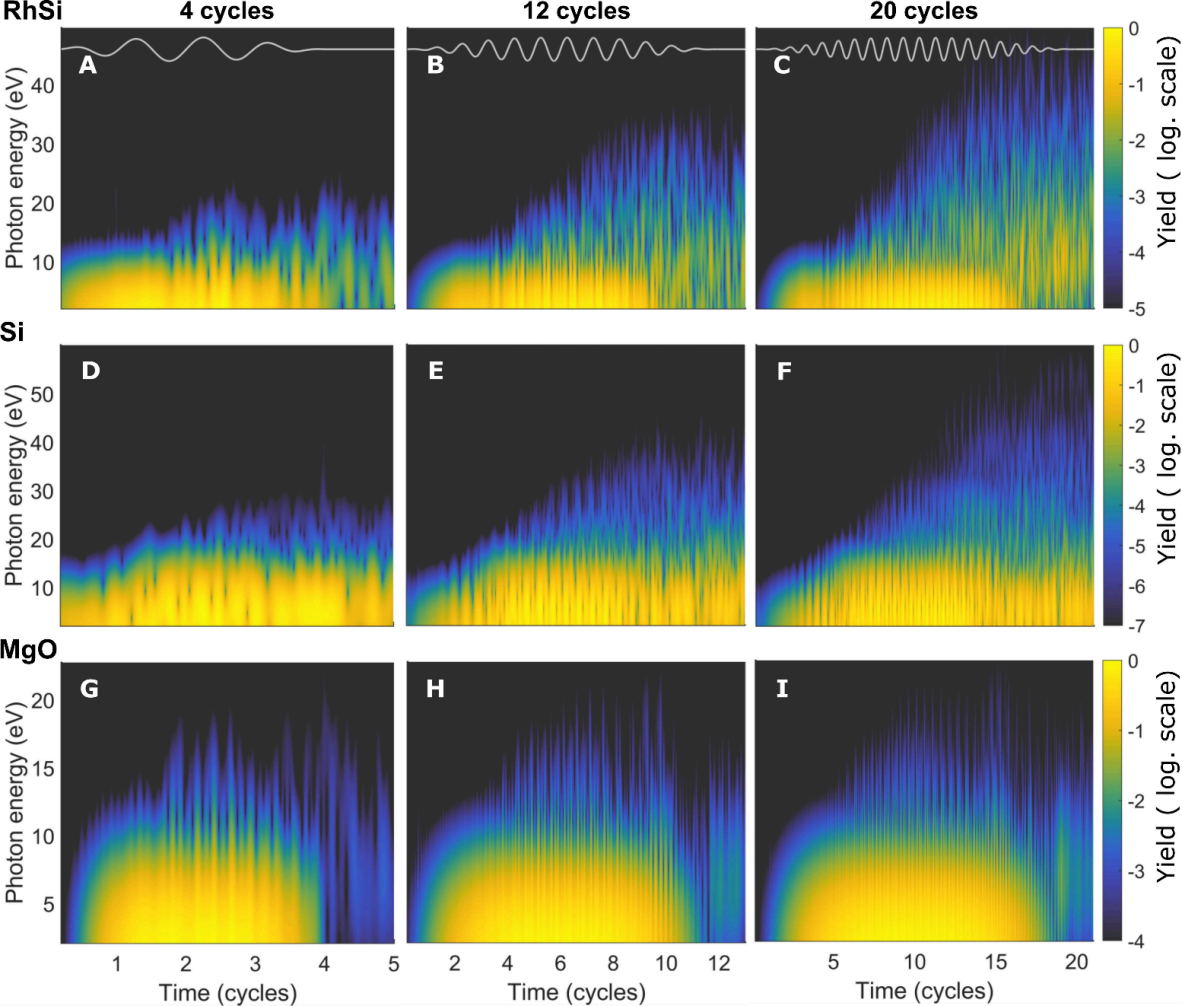
Time-resolved spectrogram of the high harmonic emission in RhSi, Si and MgO. The emission of high photon energies requires long interaction times in RhSi and Si. In both crystals, the energy cutoff increases with the interaction time. In contrast, the emission in MgO reaches similar photon energies independently of the pulse duration. The maximum yield of each time-resolved spectrogram is normalized to unity for visualization purposes. The driving pulses of 4 (left), 12 (center) and 20 optical cycles (right) are shown in gray lines at the top.

As shown by our TDDFT calculations, the duration of the laser-matter interaction determines the excited states that electron pathways can reach, and typically, the excited electron population increases during every laser cycle. The optimal duration for extending the cutoff needs to be experimentally investigated, looking for a trade-off between electron excitation and decoherence while satisfying macroscopic phase-matching conditions. Our laser parameters are similar to those of the first experiment on HHG in a nonchiral Weyl semimetal,^11^ which can serve as a reference point for experiments in chiral Weyl semimetals. Also, similar driving parameters have been employed in experiments on Dirac semimetals, such as graphene.^78^

Despite not accounting for macroscopic propagation or lattice dynamics in our simulations,^79^ the fundamental features of HHG —such as the polarization selection rules and the emergence of higher energy plateaus— are primarily governed by electron dynamics in a frozen crystal lattice.^19^ In the Supporting Information (SI),^79^ we provide additional time-dependent density-matrix results using a 1D periodic potential model including phenomenological dephasing, which allows us to assess the robustness of the pulse-duration-sensitive HHG extension to typical experimental conditions. Within this model, the pulse-duration-sensitive HHG cutoff extension is resilient to dephasing times longer than a driving cycle. Assuming a similar behavior for real materials (such as RhSi or Si), the HHG extension would survive dephasing times longer than 4 fs (for a driving photon energy of 1 eV), which is well under the electronic coherence time of 22 fs measured in graphene (another semimetallic system),^80^ and should also survive even shorter dephasing times recently predicted.^81^ Besides, the reduction of the HHG cutoff for dephasing times of half a driving cycle (see Figure S2 in SI^79^) is another important signature that interband coupling is at the origin of the HHG extension. Theoretically, even higher cutoff energies can be obtained in RhSi and Si by increasing the driving peak intensity to 3 TW/cm^2^ (see SI^79^). However, the higher level of complexity and the interferences arising from the energy broadening of the quantum pathways associated with the time-resolved spectrograms in Figure S1 compared to Figure 2 suggest an overdriven regime, which increases the risk of material damage.

In the following, we examine the dynamical symmetries of HHG^50,82^ in RhSi aiming for attosecond chiral light synthesis. For a linearly polarized driver along the chiral crystal axis [111], the emission is linearly polarized along the same direction. To exploit the chirality of the crystalline structure in HHG, we change the driving polarization to circular. Figure 3 shows the HHG and the synthesized electric field for two different crystal orientations. We set a driving photon energy of 1.55 eV, a full-pulse duration of 8 cycles, and a peak intensity of 1 TW/cm^2^.

**Figure 3:**
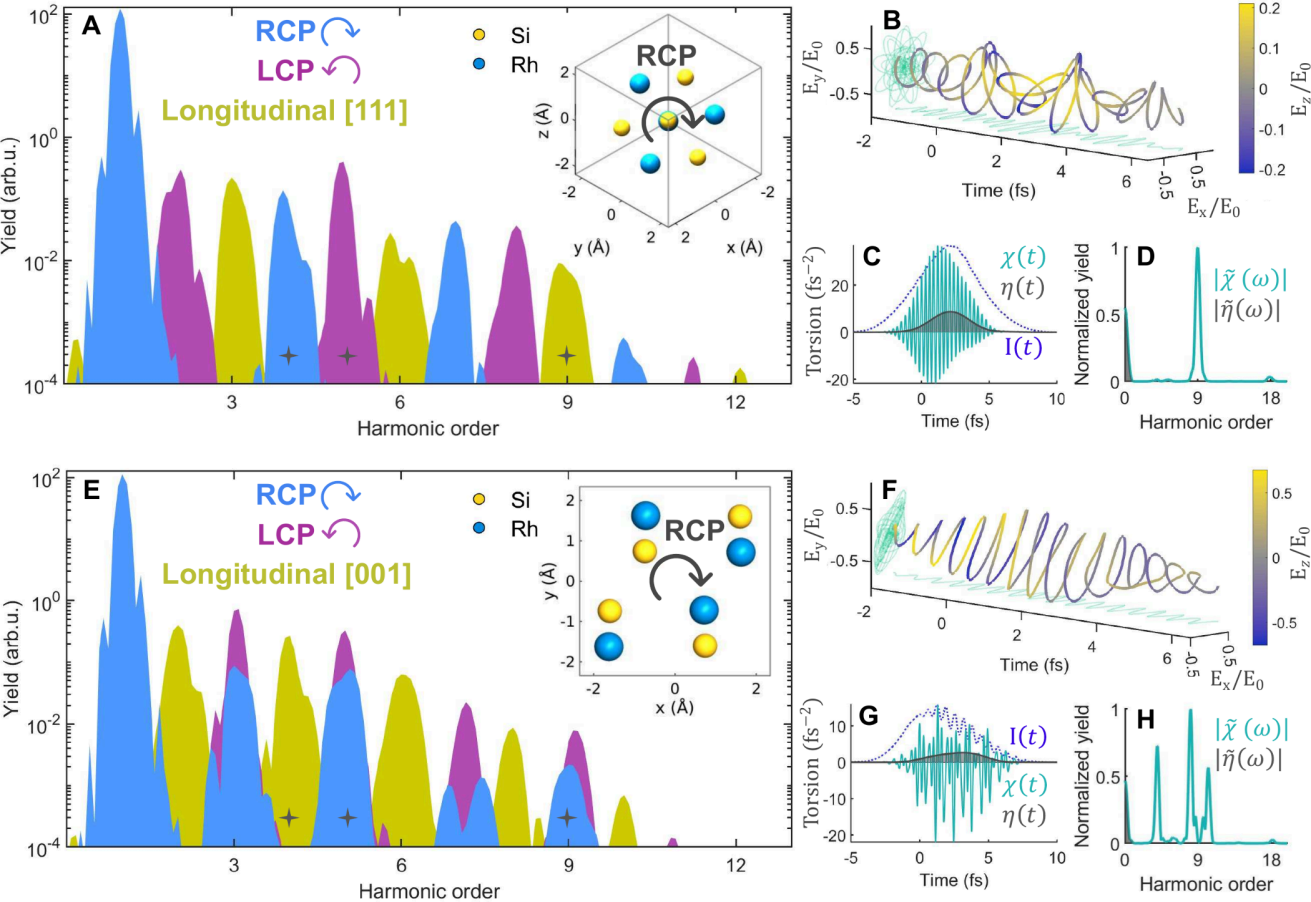
Chiral high harmonic generation in RhSi driven by a RCP pulse. The interaction of RhSi with a right circularly polarized (RCP) driver results in a complex emission with in-plane and out-of-plane polarization components. (A) The high harmonic spectrum driven by a RCP pulse in the (111) crystal plane contains alternate harmonic orders of counterrotating circular polarization (RCP in blue and LCP in violet) followed by a longitudinal component (yellow). The interaction geometry is represented in the inset. The harmonic orders 4, 5, and 9 selected for the spectral synthesis are indicated with gray stars. (B) Time evolution of the 3D synthesized electric field, with projected views plotted in light blue as a reference. Its local chirality is quantified by the instantaneous torsion (light blue) and the torsion envelope (shaded area) plotted in (C) time and (D) frequency domains. The dotted line represents the electric field intensity, *I*(*t*), in arbitrary units as a reference. (E–H) Equivalent plots are shown for a RCP driving laser pulse in the (001) crystal plane.

If a right circularly polarized (RCP) driving laser pulse propagates along the chiral axis [111], the high harmonic spectrum (Figure 3A) shows triplets of harmonic orders with the same helicity (RCP), opposite helicity —i.e. left circular polarization (LCP)— and longitudinal polarization. This selection rule is rarely found in HHG, since it requires a 3-fold rotational symmetry in tandem with a lack of mirror symmetry transverse to the rotation axis^50^ (whereas gas-phase systems are typically isotropic and therefore exhibit the mirror plane). We denote it by *C*_3_^(±)^, where (±) labels the right- and left-handed enantiomorphs associated with the crystal lattice. The absence of inversion symmetry and mirror planes is a direct feature of the B20 cubic chiral crystal structure (space group 198), enabling the emergence of longitudinally polarized harmonics (the 3*n* components in the triplets of integer *n*).

The longitudinally polarized harmonics cannot propagate in free space as plane waves, since Gauss law (***∇*** ·**E** = 0) imposes the transversality condition, **k**·**E** = 0, on the propagation direction and electric field. In the SI,^79^ we estimate the decay length of evanescent out-of-plane harmonics to be in the order of 10–100 nm, depending on the numerical aperture of the setup. Inspired by the detection of other evanescent fields, the characterization of out-of-plane harmonics can employ the principles of near-field scanning optical microscopy, where the evanescent field is transformed into propagating light that can be collected at the far-field,^83^ or chiral flux spectroscopy, which has been successfully applied to the characterization of chiral near fields from a chiral metallic nanostructure.^84^ Alternatively, by scanning the time delay of a noncollinear probe pulse, it should be possible to retrieve a pump–probe trace for the characterization of attosecond evanescent fields. Despite the detection intricacy, different evanescent fields have already been used for imaging and spectroscopic applications,^85–87^ which brings optimism for the application of attosecond near fields.

The microscopic HHG in RhSi shows a 3D-polarized electric field with both the propagating components (in-plane polarization) and the evanescent out-of-plane component. The harmonic triplets resulting from the dynamical symmetry *C*_3_^(+)^ in Figure 3A provide a main element for the locally chiral near-field synthesis: harmonic orders with three different orthogonal polarizations. The frequency of the longitudinally polarized harmonics can be matched with the frequency sum of the coplanar LCP and RCP harmonics to yield a nonvanishing average chirality in the electric field vector (i.e., locally chiral light).^72^ Unlike other types of chiral HHG, such as circular polarization,^54^ orbital angular momentum,^48^ spin–orbit coupling,^49^ or self-torque;^88,89^ the chirality is encoded directly in the 3D polarization of the local electric field.^72^ The electric field resulting from the coherent superposition of harmonic orders 4, 5, and 9 is displayed in Figure 3B in the temporal domain. The frequency of the longitudinal polarization (9ω_0_) is chosen to be the sum of the coplanar RCP (4ω_0_) and LCP (5ω_0_) harmonics, aiming to maximize the local chirality.^72^ The electric field is normalized, and the longitudinal component is represented with the colormap.

To quantify the light’s local chirality, we define the instantaneous time-dependent torsion

2 $$\chi \left(t\right)=\frac{E\left(t\right)}{E_{0}^{3}}\cdot \left(\frac{dE\left(t\right)}{dt}\times \frac{d^{2}E\left(t\right)}{dt^{2}}\right)$$

The pseudoscalar χ­(*t*) describes the noncoplanar twist of the trajectory traced by the electric field in time and electric-field space. χ­(*t*) evaluates the instantaneous chirality of the electric field **E**(*t*), being $E_{0}=\sqrt{\left|E_{x}{|}^{2}+\right|E_{y}{|}^{2}+|E_{z}{|}^{2}}$ the peak field amplitude. Figure 3C shows the ultrafast evolution of the instantaneous torsion (light blue line), the torsion envelope, η­(*t*) (shaded area), and the intensity of the local field, *I*(*t*) (dotted line, in arbitrary units). Notably, the torsion envelope, η­(*t*), evidence the asymmetry in the instantaneous torsion χ­(*t*). The time integral ζ = ∫χ­(*t*)*dt* indicates the net torsion carried by the electric field (in our case, ζ = 15.2 fs^–1^). While an electric field can be instantaneously locally chiral if χ­(t) ≠ 0, a net local chirality requires ζ ≠ 0. The definitions of χ­(*t*), η­(*t*) and ζ can be applied to quantify the instantaneous, period-averaged and net chirality of any local electric field, independently of the spectral components or the complexity of its temporal waveform. Unlike previous approaches based on correlation functions and the local degree of chirality,^74,90^ this enables the characterization of local chirality in the time domain.

Further information can be gained with the spectral decomposition of the instantaneous torsion (see Figure 3D). Denoting by ω_0_ the central driving frequency, the main frequency of the local electric field torsion is 9ω_0_, which corresponds to a period of 296 as. Smaller peaks are observed at 4ω_0_ and 5ω_0_ (with periods of 666 and 533 as), as expected from the synthesized harmonic orders. Besides, the torsion envelope is associated with the low-frequency peak (shaded area in Figure 3D), which emphasizes that the torsion of the electric field does not average to zero.

Figure 3E–H shows the HHG and the electric field analysis for a different crystal orientation. When the RCP laser pulse is contained in the (001) crystal plane, HHG is governed by a in-plane 2-fold rotational symmetry while still breaking transverse mirror symmetry, *C*_2_^(±)^. This restricts the in-plane components to odd harmonic orders, and longitudinal polarization to even harmonics (Figure 3E). The even harmonics present a high ellipticity because the RCP and LCP components do not exhibit the same amplitude, which is caused by the screw crystal axis [001]. A similar effect can arise in specific harmonic orders in other materials due to anomalous ellipticity behaviors originating from the bandstructure.^52^ As in the previous case, we synthesize the electric field (Figure 3F) by filtering harmonics 4, 5, and 9. The selection rule *C*_2_^(+)^ leads as well to a 3D electric field exhibiting attosecond torsion. However, the instantaneous torsion (light blue line in Figure 3G) reaches lower peak values and is more symmetric than in the *C*_3_^(+)^ selection rule (Figure 3C). The increase in the symmetry translates into a reduction of the torsion envelope and the net torsion, which in this case is ζ = – 5.8 fs^–1^. The negative sign indicates an opposite handedness in the torsion. The spectral components of the torsion (Figure 3H) show a more complex behavior, but still we observe both low and high frequency components, demonstrating that the *C*_2_^(+)^ selection rule also allows for the synthesis of attosecond locally chiral light.

In Figure 3, we filter specific harmonics to show optimal torsion envelopes in *C*_2_^(+)^ and *C*_3_^(+)^ cases. Filtering above a harmonic threshold also leads to attosecond locally chiral near fields with instantaneous torsion and a nonvanishing torsion envelope. The results are included in the SM^79^ to show that the attosecond 3D locally chiral polarization is a robust feature emerging from the chiral Weyl semimetal in the interaction with a circularly polarized driving pulse.

The 3D locally chiral polarization enables a dipolar laser-matter interaction at every spatial position, implying that matter systems are sensitive to the handedness of locally chiral light independently of the spatial scale. In the macroscopic picture, the total synthesized electric field using *C*_3_^(±)^ or *C*_2_^(±)^ selection rules is also expected to be globally chiral,^72^ since it is constituted by locally chiral electric fields with uniform handedness. Regarding the development of useful tools for attosecond chiral spectroscopy and for triggering chiral torsional dynamics, pump–probe schemes would be desirable. The comparison of the responses produced by time-delayed drivers with the same circular polarization versus counterrotating polarization could be exploited for circular dichroism measurements of chiral samples positioned as close as possible to the chiral crystal (ideally as a substrate or attached to the crystal surface, as employed in state-of-the-art techniques^91–93^ ). The circular dichroism emerging from the chiral crystal when driving with the opposite helicity (see SM^79^) can be subtracted by measuring the output field with and without the substrate. It also remains to be investigated whether the far-field detection of the propagating components can contain information about the attosecond locally chiral near field. Another interesting prospect would be the injection of the attosecond locally chiral field into another thin-layer material for chirality-induced spin selectivity.

In conclusion, our TDDFT calculations of HHG in RhSi and Si show a significant cutoff extension by increasing the driving pulse duration. We attribute this observation to the population of high conduction bands, which leads to higher electron–hole energy differences and thus, higher cutoff energies. This pathway of *excited ladder electrons* is particularly favored on the semimetal RhSi due to the large number of band crossings and the strong multiband coupling. In this material, the multiplateau extension merges into the main high harmonic plateau reaching higher photon energies, whereas the yield of the higher-order plateaus is weaker in Si and severely suppressed in MgO. The high cutoff energies obtained in the microscopic HHG emission from RhSi and Si establish an alternative route for surpassing the current limit of 50 eV measured in MgO.^47^ The optimal parameters for extending the cutoff require further experimental and theoretical investigation accounting for macroscopic propagation. Our results broaden the search for optimal HHG materials, a crucial issue for compact, coherent extreme-ultraviolet solid-state sources. Moreover, the mechanism of excited ladder electrons constrains the validity of few-band theoretical models, since they would fail to capture such high-excitation pathways in materials exhibiting strong coupling among multiple bands.

As a separate outcome, we demonstrate that the structural chirality of RhSi maps into locally chiral HHG.^72^ The longitudinally polarized harmonics are often ignored since they only arise in the absence of transverse mirror symmetry, and they do not propagate to the far field. Here, however, longitudinally polarized harmonics are crucial for engineering a 3D locally chiral polarization encoding attosecond torsion. This can be exploited for dipolar chiral detection or for chirality-induced spin selectivity through in situ pump–probe spectroscopies. We find that the *C*_3_^(+)^ dynamical symmetry, associated with a 3-fold rotational symmetry plus lack of transverse mirror symmetry, is advantageous for the locally chiral light synthesis over the *C*_2_^(+)^. The reason is that the *C*_3_^(+)^ enables a spectral separation into triplets of harmonics with orthogonal polarizations, whereas the *C*_2_^(+)^ mixes counterrotating circular polarization components in the even harmonic orders. Among the chiral crystals compatible with the *C*_3_^(+)^, the B20 cubic crystal structure (space group 198) is a special choice because it allows the emergence of multifold fermions^24^ and magnetic skyrmions.^94^ The findings of this work encourage future experiments measuring exotic attosecond light pulses, chirality, and high-energy excitation pathways via solid-state HHG. Further research is required to push the development of compact EUV sources, sensitive enantiomer detection, and ultrafast light-wave-driven electronics.

## Supplementary Material



## Notes

Methods and complementary results about (i) the HHG extension with the pulse duration for a higher driving intensity, (ii) density-matrix formalism to analyze the effect of dephasing in the HHG extension, (iii) the role of spectral filtering in locally chiral HHG, (iv) locally chiral HHG driven by a left-circularly polarized driver, (v) locally chiral attosecond field synthesis from analytical spectra, and (vi) estimation of the decay of the evanescent field of longitudinally polarized harmonics (PDF)
