# Supplementary material for: Pulse-Duration-Sensitive High Harmonics and Attosecond Locally Chiral Near Fields from a Chiral Topological Weyl Semimetal
Source: Nano Lett. 2026 Jul 20;26(30):9722–31. doi: 10.1021/acs.nanolett.6c00913 (PMC13450484; doi:10.1021/acs.nanolett.6c00913)
Supplement: Supplementary file 1 [file nl6c00913_si_001.pdf]

Supplementary Materials for
Pulse-duration-sensitive high harmonics and attosecond locally-chiral near fields from a chiral topological Weyl semimetal

Alba de las Heras*[1], Zhengwei Nie[2], Subhojit Pal[2], Umberto De Giovannini[1,2], Ofer Neufeld*[3], and Angel Rubio*[1,4]

[1]Max Planck Institute for the Structure and Dynamics of Matter, Center for Free-Electron Laser Science, Hamburg 22761, Germany
[2]Università degli Studi di Palermo, Dipartimento di Fisica e Chimica – Emilio Segrè, Palermo 90123, Italy
[3]Technion Israel Institute of Technology, Faculty of Chemistry, Haifa 3200003, Israel
[4]Center for Computational Quantum Physics, The Flatiron Institute, New York 10010, USA

*Emails: alba.de-las-heras@mpsd.mpg.de, ofern@technion.ac.il, angel.rubio@mpsd.mpg.de

This PDF file includes:

Methods
Complementary results about:
(i) the HHG extension with the pulse duration for a higher driving intensity
(ii) density-matrix formalism to analyze the effect of dephasing in the HHG extension
(iii) the role of spectral filtering in locally chiral HHG
(iv) locally chiral HHG driven by a left-circularly polarized pulse
(v) locally chiral attosecond field synthesis from analytical spectra
(vi) estimation of the decay of the evanescent field of longitudinally polarized harmonics
Figures S1 to S7

Methods

To compute the electron dynamics, we numerically solve the time-dependent Kohn-Sham equations of time-dependent density-functional theory (TDDFT) using the OCTOPUS open-access code [1]. We consider the adiabatic local-density approximation in the exchange-correlation functional, norm-conserving pseudopotentials, and the velocity gauge of the dipole Hamiltonian. This approach has provided accurate comparison with experimental results in solid-state HHG [2, 3].

The lattice parameters of RhSi, Si and MgO are respectively set to the experimental values 4.69, 5.44, and 4.19 Å. For the convergence of the results in RhSi and Si, we choose a $16 \times 16 \times 16$ k-point grid, spatial spacing $\Delta x = \Delta y = \Delta z = 0.40$ atomic units (a.u.), and time step $\Delta t = 0.3$ a.u. The calculations in MgO require a finer k-point sampling of the Brillouin zone $28 \times 28 \times 28$, spacing $\Delta x = \Delta y = \Delta z = 0.30$ a.u. and time step $\Delta t = 0.1$ a.u. The bandgap obtained in the DFT calculations of MgO is 4.96 eV, which is known to be substantially lower than the experimentally reported value of 7.83 eV [2, 4]. For Si, we obtain indirect and direct bandgaps of 0.50 and 2.55 eV.

The driving laser pulse is described with the vector potential

$$\mathbf{A}(t) = A_0 \sin\left(\frac{\pi t}{\tau_{\text{pulse}}}\right)^{\frac{3\left|\frac{\pi(t)}{\tau_{\text{pulse}}}-\frac{\pi}{2}\right|}{4}} \cdot \Theta\left(\frac{\tau_{\text{pulse}}}{2} - |t - \frac{\tau_{\text{pulse}}}{2}|\right) \cos(\omega_0 t)\mathbf{u_p} = A_{env}(t) A_0 \cos(\omega_0 t)\mathbf{u_p}, \tag{S1}$$

being $\mathbf{u_p}$ the unitary polarization vector, τ_{pulse} the pulse duration, ω_0 the central frequency and $A_{env}(t)$ the vector potential envelope [5]. Then, the high harmonic spectrum is computed from the time-dependent current $\mathbf{j}(t) = \sum_{\nu=x,y,z} j_\nu(t)\mathbf{u}_\nu$ as

$$|\text{HHG}(\omega)|^2 = \sum_{\nu=x,y,z} \left| \int_{-\infty}^{\infty} \frac{dj_\nu(t)}{dt} e^{-i\omega t} A_{env}(t) dt \right|^2, \tag{S2}$$

where the envelope function is included to avoid numerical artifacts from a nonvanishing signal after the laser pulse. The time-resolved spectrograms are obtained via a Gabor analysis:

$$\mathbf{S}(\omega, t) = \int_{-\infty}^{\infty} \mathbf{a}(\Omega)\, e^{-\dfrac{(\Omega-\omega)^2}{2\sigma^2}} e^{i\Omega t} d\Omega, \tag{S3}$$

using the Fourier transform of the time derivative of the current

$$\mathbf{a}(\Omega) = \int_{-\infty}^{\infty} \frac{d\mathbf{j}(t)}{dt} e^{-i\Omega t} dt, \tag{S4}$$

and setting $\sigma = 2.5\omega_0$. The synthesized electric field results from a selective filtering of specific harmonics into $\mathbf{HHG_{sel}}(\omega)$ and performing the inverse Fourier transform:

$$\mathbf{E}(t) \sim \int_{0}^{\infty} \mathbf{HHG_{sel}}(\omega) e^{i\omega t} d\omega. \tag{S5}$$

HHG extension with the pulse duration for a higher driving intensity

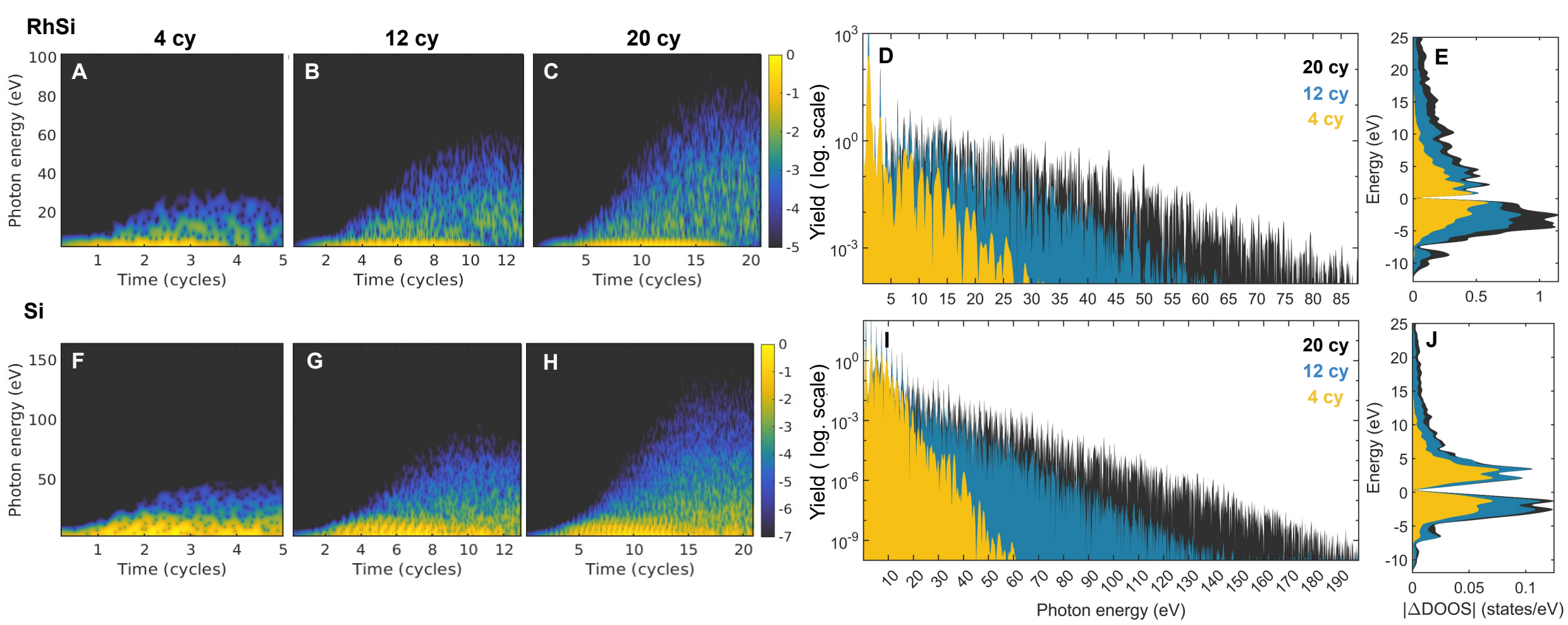

Figure S1: **Pulse duration dependence of HHG in Si and RhSi for a higher driving intensity.** Computed time-resolved spectrograms (left), high harmonic spectrum (center), and change of the density of occupied states (right).

By setting a higher driving intensity of 3 TW/cm^2, the HHG in RhSi and Si extends to higher photon energies (Fig. S1). The occupation analysis reveals a greater excitation to high conduction bands (Figs. S1E,J).

The time-resolved spectrograms (Figs. S1A-C,F-H) still present a linear increase of the HHG cutoff energy with the interaction time, while showing more interference signatures due to the larger number of HHG pathways involved. High-energy cutoffs of 80 and 180 eV are obtained in our calculations of RhSi and Si. However, the higher complexity of the HHG pathways associated with the time-resolved spectrogram of Fig. S1 compared to Fig. 2 in the main text points to an overdriven regime, increasing the risk of damage threshold.

By defining the excitation number as the ratio between the number of excited electrons and the total number of electrons in the unit cell, $n_{ex} = N_{ex}/N_e$, we quantify the excitation in the TDDFT calculations. For the cases presented in Figs. 1 and 2 of the main text, the excitation numbers after the 20-cycle driving pulse (with FWHM duration of 29.0 fs) are $n_{ex}^{(RhSi)} = 0.0870$, $n_{ex}^{(Si)} = 0.0277$ and $n_{ex}^{(MgO)} = 0.0001$, whereas Fig. S1 corresponds to $n_{ex}^{(RhSi)} = 0.2663$ and $n_{ex}^{(Si)} = 0.1480$. Thus, the results of the main text, with post-pulse excitation fractions below 9%, are expected to be below the laser-induced damage threshold. Driving intensities as high as 1.2 TW/cm^2 have been experimentally reported in Weyl and Dirac semimetals [6, 7]. Still, further experiments are required to check the limitations of the cutoff extensions.

Density-matrix formalism to analyze the effect of dephasing in the HHG extension

To assess the robustness of the HHG extension against decoherence effects, we provide in this section additional calculations in a time-dependent density-matrix (TDDM) formalism. In this approach, the electron dynamics are described using a Lindblad master equation[8, 9], where dephasing is introduced within the relaxation-time approximation (RTA)[10, 11]. The Lindblad master equation describes the time evolution of the one-particle reduced density matrix $\rho(t)$ of an open quantum system interacting with its environment:

$$\frac{d\rho(t)}{dt} = -i[H(t), \rho(t)] + \mathcal{D}[\rho(t)]. \tag{S6}$$

$H(t)$ is the time-dependent Hamiltonian of the system that gives the coherent dynamics, and $\mathcal{D}[\rho(t)]$ is the Lindblad superoperator that accounts for the dissipative processes. The Lindblad superoperator[12, 13] in Eq. (S6) can be expressed as:

$$\mathcal{D}[\rho(t)] = -\begin{pmatrix} \dfrac{\rho_{mm}(t) - \rho_{\infty,mm}}{T_1} & \dfrac{\rho_{mn}(t)}{T_2} \\ \dfrac{\rho_{nm}(t)}{T_2} & \dfrac{\rho_{nn}(t) - \rho_{\infty,nn}}{T_1} \end{pmatrix}, \tag{S7}$$

where T_1 denotes the population relaxation time and governs the decay of the diagonal density-matrix elements toward the asymptotic ground-state density matrix ρ_∞. Within the RTA, the asymptotic equilibrium density matrix is chosen as the ground-state density matrix,

$$\rho_\infty = \sum_m f_i(0) \left|\phi^{(m)}(\mathbf{r})\right\rangle \left\langle\phi^{(m)}(\mathbf{r})\right|, \tag{S8}$$

where $f_m(0)$ are the ground state occupations and $\left|\phi^{(m)}(\mathbf{r})\right\rangle$ are its corresponding orbitals. The parameter T_2 characterizes the dephasing time and regulates the decay of the off-diagonal elements of the density matrix. In realistic solid-state systems, the population relaxation time is typically much longer than the dephasing time (i.e., $T_1 \gg T_2$), since phase coherence is rapidly destroyed by elastic scattering processes before significant energy relaxation occurs. Consequently, we set $T_1 \gg T_2$ in our calculations, so that the dissipative dynamics are dominated by dephasing processes.

For simplicity, we apply this formalism to a Mathieu-type potential

$$V = -V_0(1 + \cos(2\pi x/a)), \tag{S9}$$

with lattice constant $a = 8$ a.u. and potential parameter $V_0 = 0.37$ a.u. This model has been employed in previous calculations to gain theoretical understanding of plateau extensions in solid-state HHG [14, 15]. In

Fig. S2A, we compare the TDDM results of the HHG spectra for different values of the dephasing time T_2 in the off-diagonal density matrix, governing electronic transitions. The relaxation time of the occupations (diagonal of the density matrix) is set to $T_1 = 100$ fs, on a significantly slower timescale than T_2, which is chosen to cover a range between 0.5 and 4 cycles of the fundamental frequency. The driving laser pulse is set with a photon energy of 0.39 eV, peak intensity of 0.3 TW/cm^2, and full duration of 12 cycles to observe a similar HHG extension to the TDDFT calculations in RhSi and Si (Figs. 1 and 2 of the main text). Thus, a driving cycle in the Mathieu-type potential calculations corresponds to 10.67 fs —whereas for the driving photon energy of 1 eV in Figs. 1 and 2 of the main text, the driving cycle is 4.00 fs—. The inset of Fig. S2A shows the band structure associated with the Mathieu-type potential.

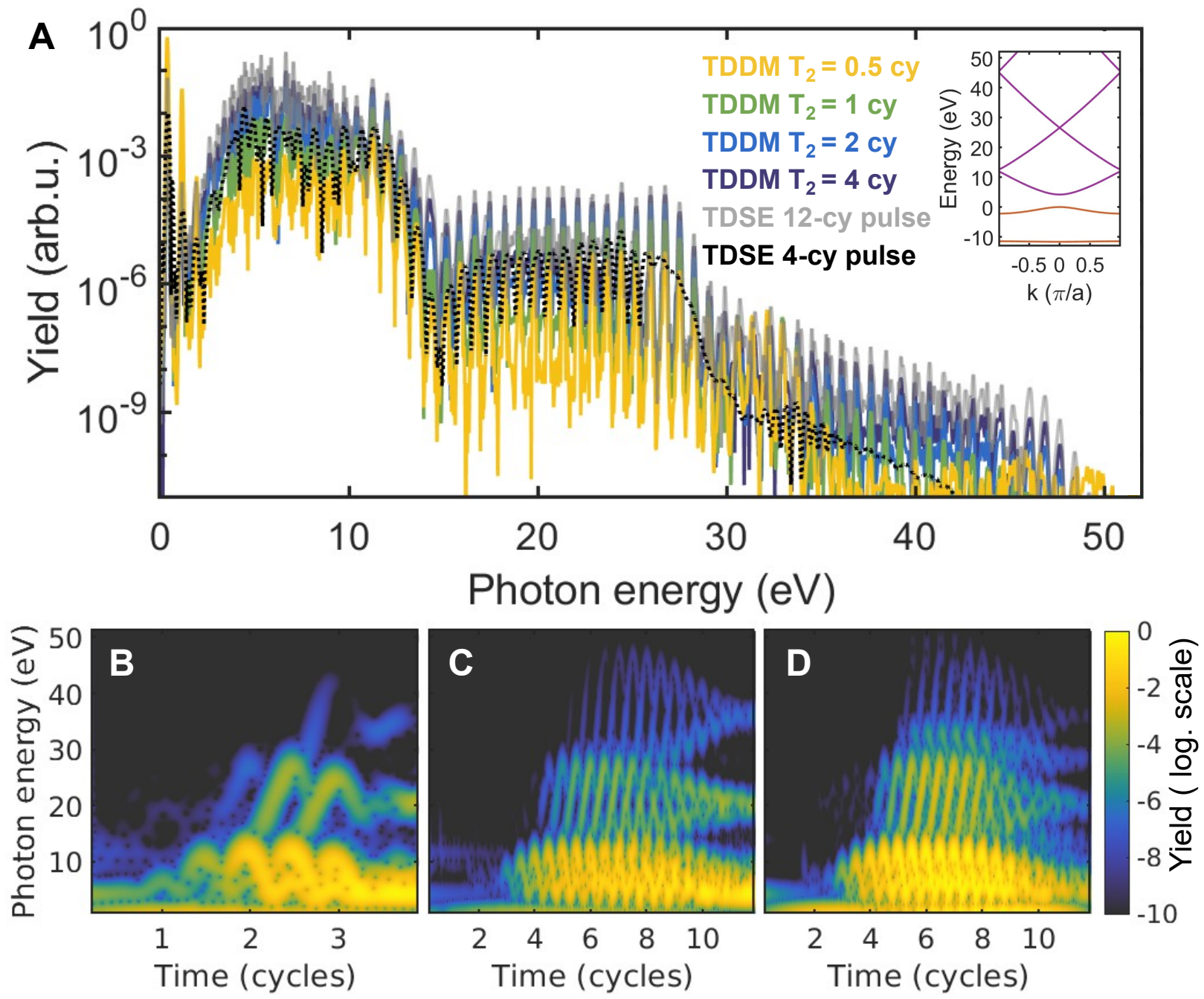

Figure S2: **Effect of the dephasing time on the pulse-duration sensitive HHG extension from a Mathieu-type potential model.** (**A**) HHG spectra comparing the results of TDDM for different dephasing times, considering a laser pulse of 12 cycles full duration, 0.39 eV driving photon energy, and 0.3 TW/cm^2 peak intensity. The analogous result obtained in the TDSE is plotted in a grey line, whereas the case of a shorter pulse of 4 cycles is displayed for comparison (black dotted line). The band structure using the Mathieu-type potential is shown in the inset. The bottom row shows the normalized time-resolved spectrograms for the following cases: (**B**) 4-cycle driving pulse in TDSE, (**C**) 12-cycle driving pulse in TDSE and (**D**) 12-cycle driving pulse in TDDM with a dephasing time $T_2 = 1$ driving cycle.

Figure S2A also shows the numerical results of the time-dependent Schrödinger equation (TDSE) for driving pulses of 4 cycles (black dotted line) and 12 cycles (grey line), while keeping constant the other driving parameters. The corresponding time-resolved spectrograms are shown in Figs. S2B and C, respectively. The TDSE results manifest a significant extension of HHG to higher photon energies with the longer driving pulse, from 26 to 47 eV. Interestingly, the HHG extension is also clearly appreciated in TDDM for dephasing times of 1 driving cycle or longer. The primary effect of dephasing is an overall reduction of the harmonic yield at all energy ranges. The high-energy region of the HHG spectra (from 33 to 47 eV) is only suppressed for dephasing times faster than half a driving cycle, which should be a feasible constraint for the experimental observation of the pulse-duration sensitive HHG extension in solid-state targets. Indeed, for the dephasing time of a half a driving cycle, the HHG cutoff takes an intermediate value around 33 eV. This demonstrates that the photon energies beyond this value are necessarily arising from interband transitions, which are the ones affected by the dephasing. This can be interpreted as another signature supporting that the HHG extension arises from excited ladder electrons requiring the coupling to high-energy conduction bands.

For a full comparison, the time-resolved spectrogram obtained in TDDM for a dephasing time of 1 cycle is shown in Fig. S2D. The main difference with the TDSE results is a harmonic plateau from 30 to 33 eV, whose yield ratio with respect to the primary plateau is enhanced. This intermediate harmonic plateau was only suppressed by setting $T_1 = T_2$. Overall, our results suggest that decoherence and relaxation processes can influence the relative yields in HHG, but should not constitute an obstacle for detecting plateau extensions in experiments of solid-state HHG.

Role of spectral filtering in locally chiral HHG

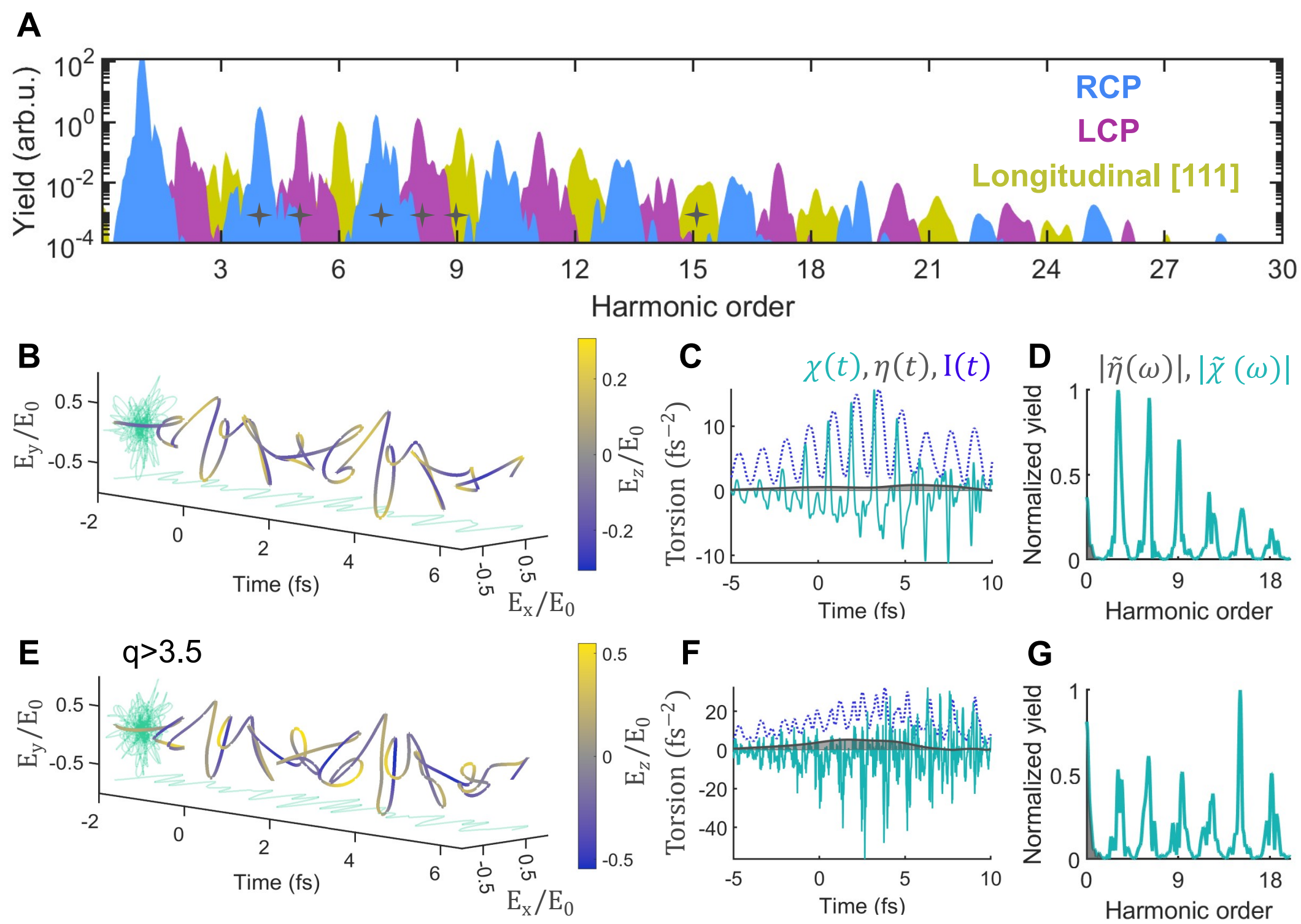

Figure S3: **Analysis of the spectral selectivity in the synthesis of the attosecond locally chiral field.** (**A**) Full high harmonic spectrum in the $C_3^{(+)}$ selection rule, considering a RCP driving pulse of 1 eV photon energy, 12 cycles full duration, and 1 TW/cm^2 peak intensity. (**B**) Local electric field resulting in the synthesis of starred harmonics, and its corresponding torsion in (**C**) time and (**D**) frequency domain. (**E-G**) Analogous plots are shown when filtering beyond the 3rd harmonic (q>3.5).

High control in the spectral filtering enables further customization of the locally chiral light and optimization of the encoded torsion. However, we should remark that it is possible to synthesize attosecond locally chiral light by just filtering above a harmonic threshold, which is a more standard procedure. Figure S3 compares the filtering of (B-D) a careful selection of multiple triplets or (E-G) beyond a threshold of $3.5\omega_0$ in the $C_3^{(+)}$ selection rule, considering a right circularly polarized (RCP) driving pulse of $\hbar\omega_0 = 1$ eV photon energy, 12 cycles full duration, and 1 TW/cm^2 peak intensity. The harmonics selected in the spectral filtering of (B-D) to optimize the electric field torsion are indicated with grey stars in (A). The selection criterion is to choose two triplets where the longitudinal harmonic is the sum of coplanar harmonics. Whereas the 3D polarization structure of the synthesized electric field is qualitatively similar, the spectral filtering selectivity enables finer tunability of the temporal waveform of the instantaneous electric-field torsion. Figure S3(C) illustrates sharply defined torsion peaks of roughly 200 as. Filtering above a frequency threshold leads to a more intricate torsion profile (Fig. S3F). Still, both filtering options show a nonvanishing instantaneous torsion and torsion envelope, demonstrating that local chirality is not emerging from a specific filtering choice,

but as a general outcome of the absence of mirror symmetry in the laser-driven interaction with RhSi.

As another example, we show in Fig. S4 the resulting electric field by filtering above a threshold of $3.5\omega_0$ in the spectra of Fig. 3A,E in the main text. The top and bottom rows correspond to the dynamical symmetry $C_3^{(+)}$ and $C_2^{(+)}$, respectively. In this filtering approach, the $C_3^{(+)}$ dynamical symmetry is again advantageous for higher values of the torsion envelope, and thus higher net chirality. This is expected due to the spectral separation of the counterrotating circular components in the $C_3^{(+)}$ case.

From the comparison of Figs. S4A-C with Figs. S3E-G, we see that the concrete values of the torsion are highly dependent on the driving parameters (such as the wavelength or pulse duration), which substantially modify the HHG spectra. Nevertheless, the polarization selection rules in HHG, which are the key element for imprinting local chirality in the electric field, are governed by the dynamical symmetry of the laser-matter interaction. Therefore, the crucial element for the locally chiral light synthesis in RhSi is the circularly polarized driver.

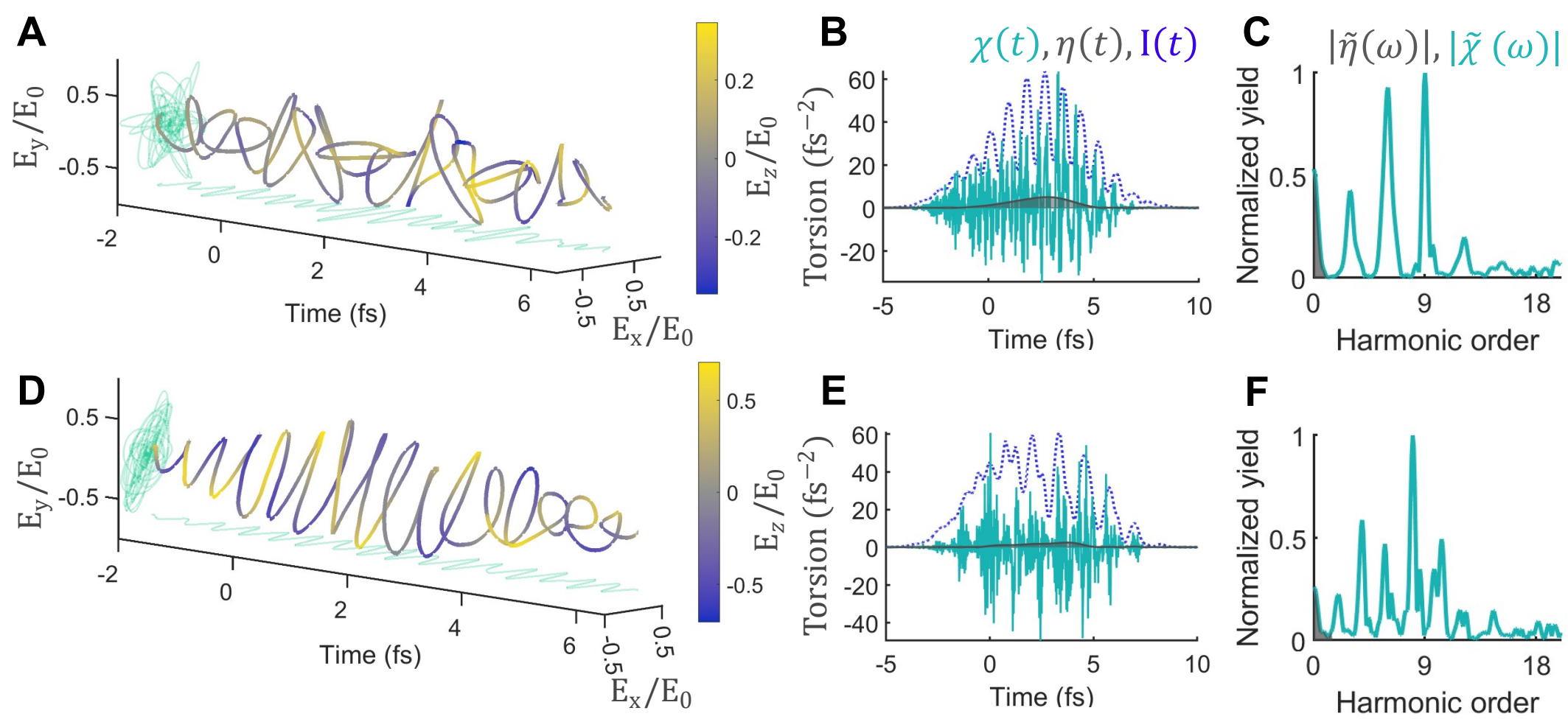

Figure S4: **Spectral filtering beyond a harmonic threshold in the synthesis of the attosecond locally chiral field.** (**A**) Local electric field resulting from filtering beyond the 3rd harmonic in Fig. 3A of the main text, and its corresponding torsion in (**B**) time and (**C**) frequency domain. (**D-F**) Analogous plots are shown for the HHG spectrum in Fig. 3E of the main text, corresponding to the $C_2^{(+)}$ dynamical symmetry.

Locally chiral HHG driven by a left-circularly polarized pulse

In Fig. S5, we set the driving helicity opposite to the handedness of the chiral crystal structure. The other laser parameters are the same as Fig. 3 in the main text. The HHG presents a highly dichroic response if the driving field is contained in the (111) plane, orthogonal to the chiral crystal axis (Fig. S5A). Not only is the helicity of the harmonics reversed, but we also notice substantial differences in the harmonic spectral shape. The circular dichroism, defined as the absolute value of the difference in the HHG spectra originated from a RCP and LCP driver $||HHG^{(\text{RCP driver})}(\omega)|^2 - |HHG^{(\text{LCP driver})}(\omega)|^2|$, is plotted as a solid grey line. The case of a RCP driver is also displayed as a dotted line to facilitate the comparison.

The circular dichroism manifests as a strong modification in the light synthesis (Fig. S5B), where the asymmetry of the instantaneous torsion $\chi(t)$ is reduced and shows a more complex behavior (Fig. S5C). The peak of the envelope torsion in $\eta(t)$ and the net torsion $\zeta = 2.7$ fs^{-1} are also diminished. The differences are also manifested in the frequency domain of the torsion (Fig. S5D). On the contrary, for a driver contained in the (001) crystal plane, the high harmonic spectrum is very similar for RCP and LCP drivers, except for a flip in the helicity of the harmonics (Fig. S5E). The same torsion is obtained in Figs. 3F,H, and Figs. S5F,H. For the crystal orientation (001), HHG does not sense the handedness of the crystal, but it emerges when the propagation direction is contained along the chiral crystal axis [111].

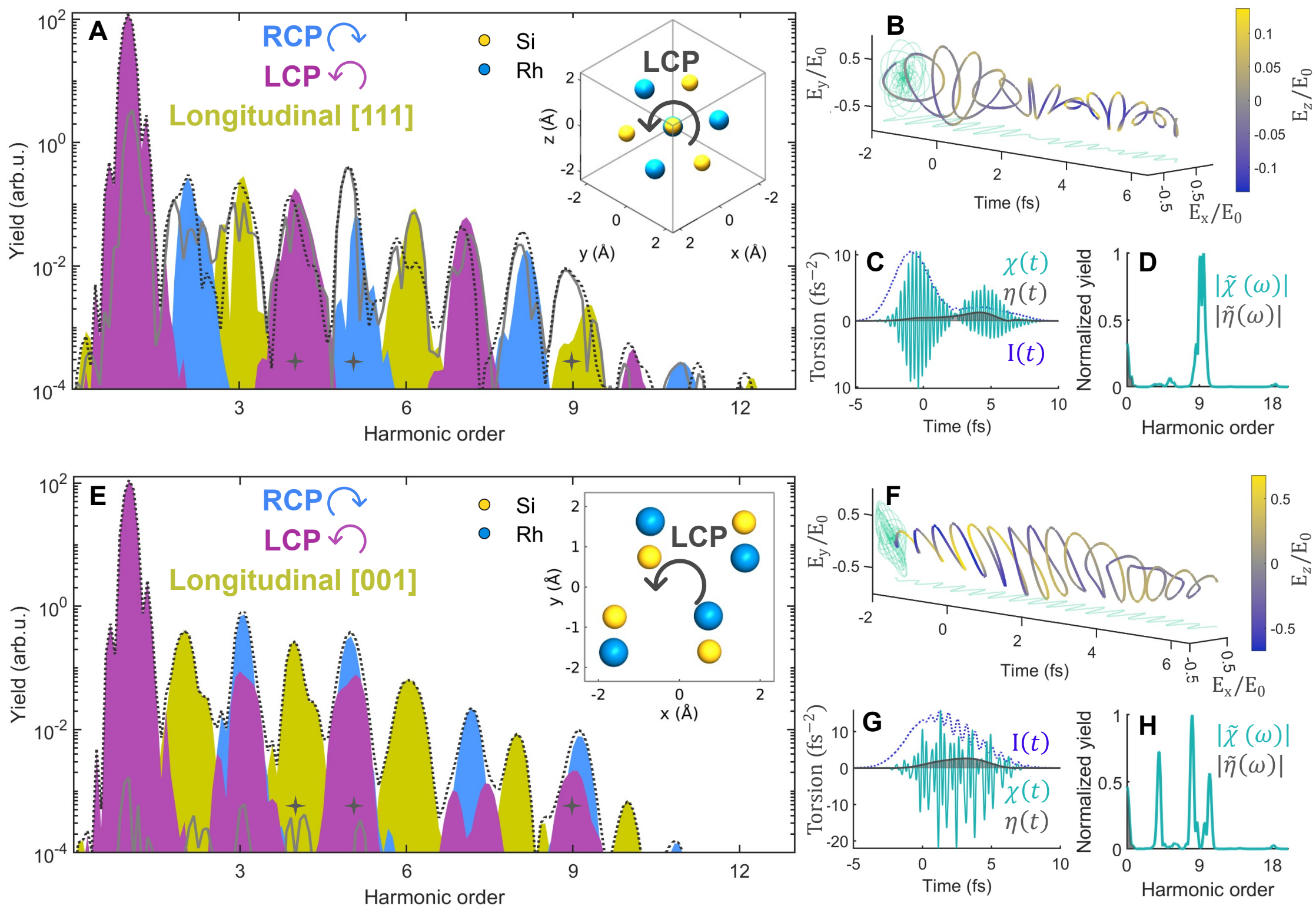

Figure S5: **Chiral high harmonic generation in RhSi driven by a LCP pulse.** Equivalent plots to Fig. 3 considering opposite driving helicity. The circular dichroism (solid line) and the case of a RCP driver (dotted line) are superimposed in (**A**) and (**E**) panels to highlight the differences in the high harmonic spectra driven from a RCP or LCP driver. The circular dichroism is maximized when the propagation direction matches the chiral axis of the crystal [111].

Locally chiral attosecond field synthesis from analytical spectra

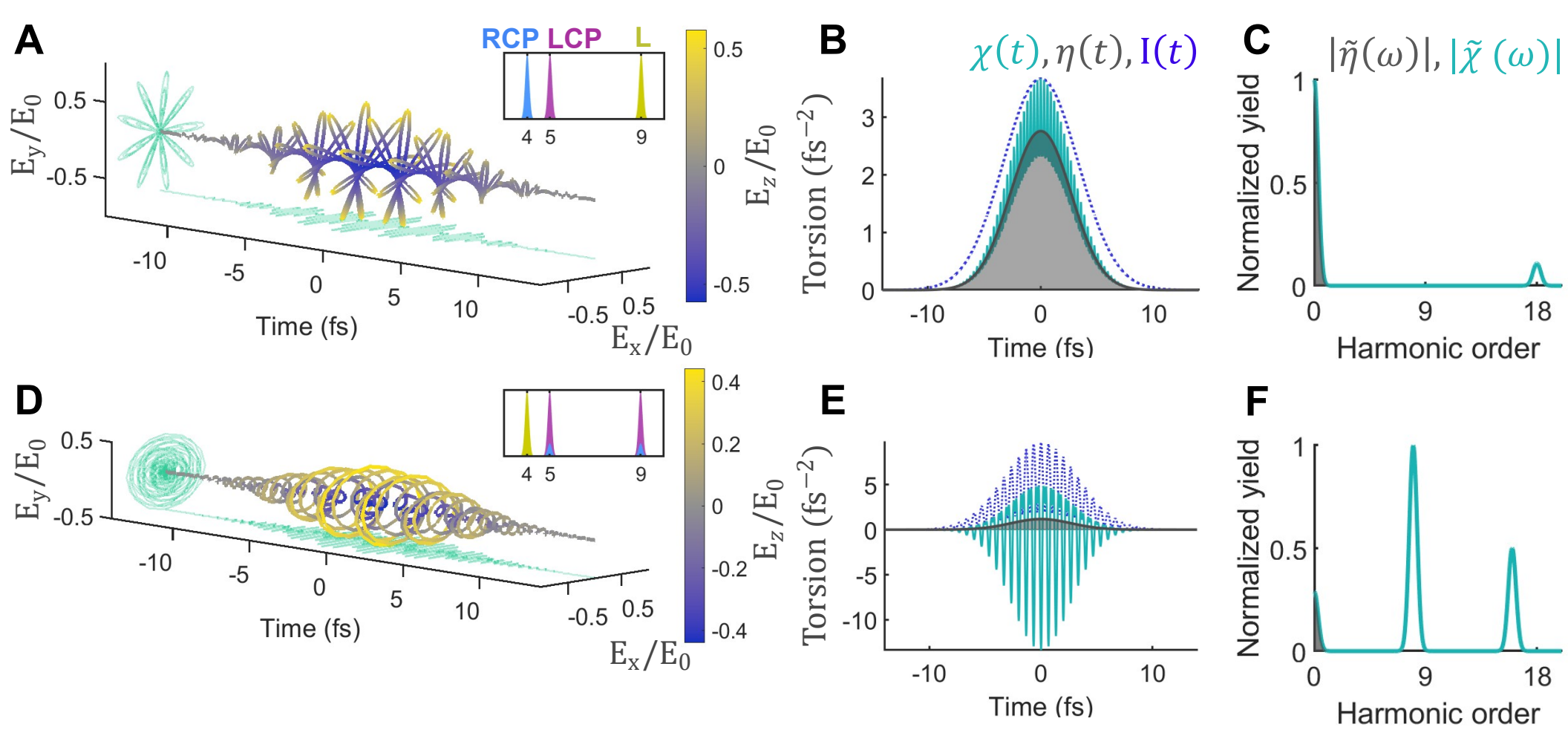

Figure S6: **Attosecond locally chiral light from a harmonic triplet in analytical spectra.** (**A**,**D**) Local electric field resulting from the harmonic triplet shown in the inset. The corresponding torsion in (**B**,**E**) time and (**C**, **F**) frequency domain. $C_3^{(+)}$ (top) and $C_2^{(+)}$ (botom) dynamical symmetries are considered.

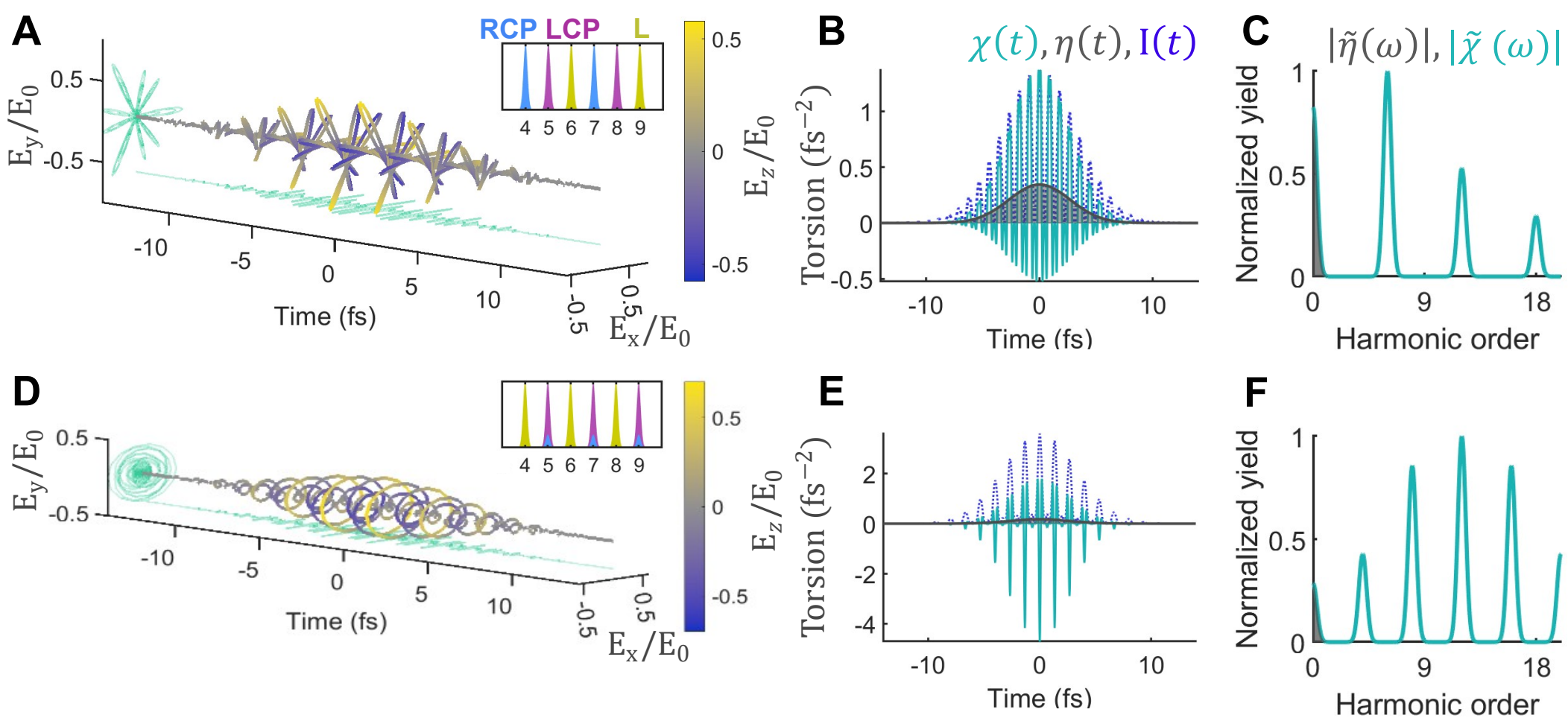

Figure S7: **Attosecond locally chiral light by filtering beyond a harmonic threshold in analytical spectra.** (**A**,**D**) Local electric field resulting from the spectrum shown in the inset, covering harmonic orders from 4 to 9. The corresponding torsion in (**B**,**E**) time and (**C**, **F**) frequency domain. $C_3^{(+)}$ (top) and $C_2^{(+)}$ (botom) dynamical symmetries are considered.

In the complementary results of this section, we obtain the locally-chiral attosecond field from an analytical spectrum constituted by a sum of Gaussian functions. The motivation of this analysis is to understand the locally chiral light synthesis in an ideal, simplified picture in which all the harmonics have the same amplitude and spectral profile. In the ideal triplet with equal-amplitude harmonics following the $C_3^{(+)}$ selection rule (first row in Fig. S6), the instantanous torsion is asymmetric at all times (only presenting positive values in Fig. S6B), and the torsion envelope follows the pulse envelope. Thus, a similar harmonic yield and spectral shape is desirable in the harmonics triplets of $C_3^{(+)}$ to maximize the local chirality. On the other hand, the harmonic triplet of $C_2^{(+)}$ requires a different amplitude in the RCP and LCP components to synthesize a locally chiral field, since otherwise the total electric field would lie in a plane. The main difference of the analytical scenario (second row in Fig. S6) compared to the HHG numerical simulations (Fig. 3F-H) is a more defined and regular helicity in the coplanar components. The instantaneous torsion in Fig. S6E displays sharp periodic peaks of short duration modulated by the pulse envelope. The asymmetry of the instantanous is also enhanced towards negative values in the ideal analytic case compared to the HHG simulations.

The filtering of six consecutive harmonics in the analytical spectra of Fig. S7 leads again to simpler torsion profiles than in the HHG simulations. A trivial difference is that the analytic results in Figs. S6 and S7 follow the pulse envelope, whereas the HHG simulations (Fig. 3 of the main text and Fig. S4) present a shift to later times after the peak envelope. At the instant of the peak envelope, the population of valence bands becomes more depleted, so that more quantum HHG pathways are initiated. This intuitively explains the delay in the maximum yield of HHG, and accordingly in the encoded electric-field torsion resulting from the quantum simulations.

In the $C_3^{(+)}$ case (top row in Fig. S7), the torsion asymmetry is reduced when using broad filtering instead of isolating a harmonic triplet. The instantanous torsion in Fig. S7B is no longer purely restricted to positive values. We also note that the peaks in the instantaneous torsion of $C_2^{(+)}$ (Fig. S7E) become more separated in time as the number of filtered harmonics increases. These trends are more complex in the results from strong-field interactions, where amplitude mismatches and relative phase differences across harmonics also influence the synthesis of attosecond pulses.

Estimation of the decay of the evanescent field of longitudinally polarized harmonics

The synthesis of locally chiral light requires the longitudinally polarized harmonics produced in the nonlinear interaction of a circularly polarized driver with RhSi. In this section, we aim to provide an estimation of the decay of the evanescent field in free space after the boundary of the chiral crystal ($z > 0$). Since the different frequency components of the electromagnetic field can be decoupled in the free-space propagation, we derive the condition imposed by the Helmholtz equation

$$\nabla^2 E_z(x, y, z) + k_{q,0}^2 \, E_z(x, y, z) \tag{S10}$$

to a longitudinally polarized field, $E_z(x, y, z)$, corresponding to harmonic order q. The harmonic wavelength $\lambda_q = \lambda_0/q$ and magnitude of the wavevector $k_{q,0} = 2\pi/\lambda_q = 2\pi q/\lambda_0$ are written in terms of the fundamental driving wavelength λ_0. By considering the harmonic field as a monochromatic plane wave mode

$$E_z(x, y, z) = E_{z,0} e^{i(k_{q,x}x + k_{q,y}y + k_{q,z}z)}, \tag{S11}$$

the transversal and longitudinal wavevector components are related by

$$k_z = \sqrt{k_q^2 - k_{q,\perp}^2} = \sqrt{k_q^2 - k_{q,x}^2 - k_{q,y}^2}. \tag{S12}$$

Equation (S12) establishes that the harmonic field propagates if $k_{q,\perp} < k_q$. On the contrary, if $k_{q,\perp} > k_q$, the longitudinal wavevector component is an imaginary number $k_{q,z} = i\alpha_q$ and the harmonic field is evanescent:

$$E_z(x, y, z) = E_{z,0} e^{i(k_{q,x}x + k_{q,y}y)} e^{-\alpha_q z}. \tag{S13}$$

The numerical aperture (NA) of the setup, which is the parameter characterizing the emission angles of the longitudinal harmonic field, determines the transversal wavevector component

$$k_{q,\perp} = k_q \text{ NA}. \tag{S14}$$

Thus, the attenuation coefficient becomes

$$\alpha_q = \sqrt{k_{q,\perp}^2 - k_q^2} = k_q \sqrt{\text{NA}^2 - 1}. \tag{S15}$$

The distance at which the longitudinal harmonic field drops to $E_{z,0}/e$ is the decay length of the evanescent field:

$$\delta_q = \frac{1}{\alpha_q} = \frac{\lambda_0}{2\pi q \sqrt{\text{NA}^2 - 1}}. \tag{S16}$$

For the 9th harmonic in the $C_3^{(+)}$ selection rule of Fig.3A in the main text, numerical apertures of 1.01, 1.1, 1.5 and 2, are respectively associated with decay lengths of 99.8, 30.9, 12.7 and 8.2 nm. As another illustrative case, the 4th harmonic in the $C_2^{(+)}$ selection rule of Fig.3E in the main text, numerical apertures of 1.01, 1.1, 1.5 and 2, are respectively associated with decay lengths of 224.5, 69.5, 28.5 and 18.4 nm. This shows that, for the detection or in-situ application of the evanescent field, the detector or sample must be extremely close to the chiral crystal. Ideally, the sample should be positioned directly at the surface of the solid, as in recent state-of-the-art spectroscopy techniques [16–18]. On the other hand, for numerical apertures less than unity, the longitudinal harmonic field could persist over longer distances determined by Gauss law ($\nabla \cdot \mathbf{E} = 0$). For this, macroscopic calculations beyond the scope of this work would be required.

References

[1] N. Tancogne-Dejean, M. J. Oliveira, X. Andrade, H. Appel, C. H. Borca, G. L. Breton, F. Buchholz, A. Castro, S. Corni, A. A. Correa, U. D. Giovannini, A. Delgado, F. G. Eich, J. Flick, G. Gil, A. Gomez, N. Helbig, H. Hübener, R. Jestädt, J. Jornet-Somoza, A. H. Larsen, I. V. Lebedeva, M. Lüders, M. A. Marques, S. T. Ohlmann, S. Pipolo, M. Rampp, C. A. Rozzi, D. A. Strubbe, S. A. Sato, C. Schäfer, I. Theophilou, A. Welden, A. Rubio, "Octopus, a computational framework for exploring light-driven phenomena and quantum dynamics in extended and finite systems", *Journal of Chemical Physics* **2020**, *152*, 124119, DOI 10.1063/1.5142502/954926.

[2] N. Tancogne-Dejean, O. D. Mücke, F. X. Kärtner, A. Rubio, “Ellipticity dependence of high-harmonic generation in solids originating from coupled intraband and interband dynamics”, *Nature Communications* **2017**, *8*, 745, DOI 10.1038/s41467-017-00764-5.

[3] N. Klemke, N. Tancogne-Dejean, G. M. Rossi, Y. Yang, F. Scheiba, R. E. Mainz, G. D. Sciacca, A. Rubio, F. X. Kärtner, O. D. Mücke, “Polarization-state-resolved high-harmonic spectroscopy of solids”, *Nature Communications* **2019**, *10*, 1319, DOI 10.1038/s41467-019-09328-1.

[4] R. Whited, C. J. Flaten, W. Walker, “Exciton thermoreflectance of MgO and CaO”, *Solid State Communications* **1973**, *13*, 1903–1905, DOI 10.1016/0038-1098(73)90754-0.

[5] O. Neufeld, O. Cohen, “Background-Free Measurement of Ring Currents by Symmetry-Breaking High-Harmonic Spectroscopy”, *Physical Review Letters* **2019**, *123*, 103202, DOI 10.1103/PhysRevLett.123.103202.

[6] Y.-Y. Lv, J. Xu, S. Han, C. Zhang, Y. Han, J. Zhou, S.-H. Yao, X.-P. Liu, M.-H. Lu, H. Weng, Z. Xie, Y. B. Chen, J. Hu, Y.-F. Chen, S. Zhu, “High-harmonic generation in Weyl semimetal β-WP2 crystals”, *Nature Communications* **2021**, *12*, 6437, DOI 10.1038/s41467-021-26766-y.

[7] T. Higuchi, C. Heide, K. Ullmann, H. B. Weber, P. Hommelhoff, “Light-field-driven currents in graphene”, *Nature* **2017**, *550*, 224–228, DOI 10.1038/nature23900.

[8] G. Lindblad, “On the generators of quantum dynamical semigroups”, *Communications in Mathematical Physics* **1976**, *48*, 119–130, DOI 10.1007/BF01608499.

[9] H.-P. Breuer, F. Petruccione, *The Theory of Open Quantum Systems*, *Vol. 9780199213900*, Oxford University PressOxford, **2007**, pp. 1–656, DOI 10.1093/acprof:oso/9780199213900.001.0001.

[10] G. Roósz, “Lindbladian way for the relaxation time approximation, application to Kibble-Zurek processes due to environment temperature quench, and to Lindbladian perturbation theory”, **2024**, 1–5.

[11] D. G. Tempel, A. Aspuru-Guzik, “Relaxation and dephasing in open quantum systems time-dependent density functional theory: Properties of exact functionals from an exactly-solvable model system”, *Chemical Physics* **2011**, *391*, 130–142, DOI 10.1016/j.chemphys.2011.03.014.

[12] S. A. Sato, P. Tang, M. A. Sentef, U. D. Giovannini, H. Hübener, A. Rubio, “Light-induced anomalous Hall effect in massless Dirac fermion systems and topological insulators with dissipation”, *New Journal of Physics* **2019**, *21*, 093005, DOI 10.1088/1367-2630/ab3acf.

[13] Z. Nie, S. Pal, S. A. Sato, H. Hübener, U. D. Giovannini, **2026**, in preparation.

[14] M. Wu, D. A. Browne, K. J. Schafer, M. B. Gaarde, “Multilevel perspective on high-order harmonic generation in solids”, *Physical Review A* **2016**, *94*, DOI 10.1103/PhysRevA.94.063403.

[15] T. Ikemachi, Y. Shinohara, T. Sato, J. Yumoto, M. Kuwata-Gonokami, K. L. Ishikawa, “Trajectory analysis of high-order-harmonic generation from periodic crystals”, *Physical Review A* **2017**, *95*, 043416, DOI 10.1103/PhysRevA.95.043416.

[16] D. Lüftner, T. Ules, E. M. Reinisch, G. Koller, S. Soubatch, F. S. Tautz, M. G. Ramsey, P. Puschnig, “Imaging the wave functions of adsorbed molecules”, *Proceedings of the National Academy of Sciences* **2014**, *111*, 605–610, DOI 10.1073/pnas.1315716110.

[17] H. Offenbacher, D. Lüftner, T. Ules, E. M. Reinisch, G. Koller, P. Puschnig, M. G. Ramsey, “Orbital tomography: Molecular band maps, momentum maps and the imaging of real space orbitals of adsorbed molecules”, *Journal of Electron Spectroscopy and Related Phenomena* **2015**, *204*, 92–101, DOI 10.1016/J.ELSPEC.2015.04.023.

[18] K. Baumgärtner, M. Reuner, C. Metzger, D. Kutnyakhov, M. Heber, F. Pressacco, C. H. Min, T. R. Peixoto, M. Reiser, C. Kim, W. Lu, R. Shayduk, M. Izquierdo, G. Brenner, F. Roth, A. Schöll, S. Molodtsov, W. Wurth, F. Reinert, A. Madsen, D. Popova-Gorelova, M. Scholz, “Ultrafast orbital tomography of a pentacene film using time-resolved momentum microscopy at a FEL”, *Nature Communications 2022 13:1* **2022**, *13*, 2741–, DOI 10.1038/s41467-022-30404-6.
